# Medical Students’ Attitudes Towards Cardiothoracic Surgery in the United Kingdom: A Cross-Sectional Survey of 1675 Students

**DOI:** 10.1177/23821205211072722

**Published:** 2022-01-12

**Authors:** Samiullah Dost, Lana Al-Nusair, Mai Shehab, Arwa Hagana, Aleena Hossain, Ahmed Jawad Dost, Aida Abdelwahed

**Affiliations:** 1 Guy’s and St Thomas’ NHS Foundation Trust, SE11 4TX; 2 Imperial College London, London, UK, SW7 2AZ; 3 King’s College London, London, UK, SE1 1UL; 4 University of Southampton, Southampton, UK, SO17 1BJ

**Keywords:** cardiothoracic surgery (CTS), medical education & training, education & training, thoracic surgery, transplant surgery, cardiac surgery

## Abstract

**Objectives:**

The objectives of this study were the following: (i) assess interest levels in cardiothoracic surgery (CTS) among UK-based medical students, (ii) identify potential motivators and barriers to pursuing CTS training, (iii) explore the influence of gender on interest in CTS in greater depth.

**Methods:**

Medical students from all year groups across UK medical schools were invited to participate in a cross-sectional, national online survey. Responses were collected from 02/12/2019 to 08/12/2019.

**Results:**

1675 medical students from 31 UK medical schools responded, with an estimated 5.3% response rate. Of the respondents, 33.7% respondents reported having exposure to CTS, primarily through their medical school or through extracurricular activities (48.4% and 38.8%, respectively). When assessing interest in CTS, 31.4% were interested in undertaking a career in CTS, with a larger proportion of students expressing interest with no exposure to CTS than those with exposure. However, interest in pursuing CTS decreased with exposure as medical students transitioned from pre-clinical to clinical stages. Additionally, male participants were more interested in seeking a CTS post than their female counterparts (38% *vs.* 27.6%). The length of training (*p* = 0.0009) and competitive nature (*p* < 0.0001) of gaining a CTS post were the primary deterring factor for female participants, compared to their male counterparts.

**Conclusions:**

This study shows the importance of quality of exposure and its impact on students’ interests in pursuing a career in CTS. The negative relationship between exposure and interest in CTS can be associated with the realisation of the challenges that come with pursuing CTS.

## Introduction

Cardiothoracic surgery (CTS) is the surgical management of diseases affecting the heart and thorax. With a predicted growth in world population coupled with an aging population, there is a growing need to train and retain cardiothoracic surgeons in order to meet current and future clinical demands. The UK CTS training programme consists of 8 years of speciality training (ST1-ST8). CTS training starts at ST1 level after completing 2 years of the foundation programme (FY1/FY2) post-medical school graduation. A number of ST3 places are available for applicants applying after 2 years of core surgical training post FY1/FY2.^1^

Currently, only 59% of cardiothoracic surgeons in the UK are trained in the UK and Ireland, with entries into CTS at ST3 declining at a steady rate.^2,3^ However, this is in contrast to figures from ST1 trainees which appear to show that there is an increase in ST1 applicants to the speciality from 2016 to 2018.^3^

It is important to identify the current perceptions of medical students towards the speciality in order to determine actions required to maintain speciality interest and to ensure adequate recruitment in the future. The most common factor associated with interest is speciality exposure.^4,5^ Other factors which may influence speciality interest include social pressures of the job, the changing demographic of medical students, current perceptions of cardiothoracic surgeons and the rigorous training pathway.^6^

It is important to note that these factors may differ between male students and their female counterparts and that this has not been thoroughly explored in the context of CTS. Female cardiothoracic surgeons in the UK make up only 3% of doctors within the speciality, and literature suggests that female students have different barriers due to experiences than male students; further exploration into differences in barriers and motivators between male and female medical students considering entering CTS is pivotal and must be explored.^3,7,8^

With the landscape of CTS constantly changing and the American Society of Thoracic Surgeons reporting a stagnation in the number of cardiac surgeries (perhaps due to primary prevention and better control of risk factors), it is important that a steady influx of enthusiastic and motivated junior doctors is recruited in the field, driving the evolution of CTS.^9^

This study explores medical student perceptions of CTS through three key aims; by investigating the level of interest and exposure of students to CTS, identifying potential motivators and barriers to CTS training and assessing the influence of gender on interest in the specialty.

## Material and Methods

### Survey Design and Distribution

This was a national cross-sectional study conducted via an online survey platform (Google Forms). Medical students in the UK across all year groups were invited to complete the online questionnaire from 02/12/2019 to 08/12/2019. The questionnaire was distributed by medical students via social media, to widen outreach to all UK registered medical schools. This involved posts on platforms including Facebook, Twitter, and Instagram with an anonymous link to the questionnaire. The questionnaire evaluated students’ personal future speciality interest, interest in undertaking a CTS career pathway and assessing their attitudes towards CTS. These domains were explored using a 5-point Likert scale in response to statements regarding the speciality (Appendix). The survey items were drafted, discussed with a group of medical students after which questions were reviewed and refined.

Non-response bias was minimised by ensuring the survey was distributed by many medical students of diverse backgrounds, from various medical schools and across a range of different platforms. Surgical societies and CTS societies were avoided to minimise response bias.

### Data Analysis

The data was exported from Google Forms using Microsoft Excel (Excel version 16.29, 2019). GraphPad Prism (Prism version 8.2.1, 2019) was used for data analysis. A Kruskal-Wallis test was conducted to investigate differences in CTS interest between the different year groups, followed by a post-hoc Mann-Whitney test to assess significance. To assess for significant differences in the scoring of the statements between genders, a Mann-Whitney test was conducted. All P values less than 0.05 were considered to be significant.

### Participant Consent and Ethical Considerations

Ethical approval was requested from Imperial College London. This study was deemed not to require ethical approval, as all responses were anonymised, and informed consent was given by students prior to completing the survey.

## Results

### Cohort Demographics and Interest in CTS

A total of 1675 students across all year groups from 31 medical schools responded with an estimated response rate of 5.3%, based on approximate numbers of total medical students at these medical schools (Table 1).^10^ Of the respondents, 37.0% were male (*n* = 619), 62.5% were female (*n* = 1046) and 0.5% identified as other genders (*n* = 10).

**Table 1.** A table outlining the demographics of the survey participants (*n* = 1675), including their gender, year of medical school and university.

Demographic		Proportion Of Students, % (N)
Gender	Male	37.0 (619)
	Female	62.5 (1046)
	Other	0.5 (10)
		
Year	Year 1	20.0 (335)
	Year 2	18.0 (301)
	Year 3	27.8 (466)
	Intercalated year	7.3 (122)
	Penultimate year	17.7 (296)
	Final year	9.3 (155)
		
University	University of Aberdeen School of Medicine and Dentistry	0.1 (1)
	Anglia Ruskin University School of Medicine	1.0 (16)
	Barts and The London School of Medicine and Dentistry	3.2 (54)
	University of Birmingham College of Medical and Dental Sciences	4.4 (74)
	Brighton and Sussex Medical School	1.6 (27)
	University of Bristol Medical School	1.1 (18)
	University of Buckingham Medical School	0.8 (14)
	University of Cambridge School of Clinical Medicine	0.3 (5)
	Cardiff University School of Medicine	4.8 (80)
	University of Exeter Medical School	2.9 (48)
	Hull York Medical School	0.2 (4)
	Imperial College London Faculty of Medicine	4.3 (72)
	Keele University School of Medicine	6.1 (102)
	King's College London GKT School of Medical Education	12.1 (203)
	Lancaster University Medical School	0.1 (1)
	University of Leeds School of Medicine	8.8 (147)
	University of Leicester Medical School	4.8 (81)
	University of Liverpool School of Medicine	0.2 (4)
	University of Manchester Medical School	5.7 (95)
	Newcastle University School of Medical Education	0.4 (7)
	Norwich Medical School	5.3 (89)
	University of Nottingham School of Medicine	1.7 (28)
	University of Oxford Medical Sciences Division	5.4 (90)
	Queen's University Belfast School of Medicine	2.5 (42)
	University of Southampton School of Medicine	1.0 (16)
	University of St Andrews School of Medicine	0.2 (3)
	St George's, University of London	3.3 (55)
	Swansea University Medical School	1.6 (26)
	University of Central Lancashire School of Medicine	2.7 (45)
	University College London Medical School	4.8 (80)
	University of Warwick Medical School	5.8 (97)
	Other (Unclear abbreviation eg UOB)	3.0 (51)

With regards to interest in CTS, 31.4% (*n* = 526) students stated they were considering a career in CTS, of which 73 students said it was their first choice. Most students, although not interested primarily in CTS, were interested in a wide range of specialities, including medical subspecialities (25.6%, *n* = 429), surgical subspecialities (23.9%, *n* = 400), and general practice (12.5%, *n* = 210); only 11.9% (*n* = 199) of students were undecided on a particular speciality.

The majority of students (45.1%, *n* = 237) who were interested in pursuing CTS were undecided on CTS subspeciality. Of those decided, 32.7% (*n* = 172) selected cardiac surgery as their most desired subspeciality, while 7.2% (*n* = 39) opted for congenital CTS, 6.3% (*n* = 34) transplant subspeciality, and 6.1% (*n* = 32) thoracic surgery.

### Exposure and Interest in CTS

When investigating student exposure to the CTS career pathway, 33.7% (*n* = 564) of students reported having exposure to CTS in comparison to 66.3% (*n* = 1111) who had no prior exposure. Of those with exposure, 48.4% (*n* = 273) of students had contact with the speciality primarily through their medical school, 38.8% (*n* = 219) were exposed to CTS through extracurricular activities, and 12.8% (*n* = 72) of students were exposed through a mixture of both. In particular, 44.5% (*n* = 69) of final year students reported having had exposure to CTS, with 34.2% (*n* = 53) of final years having had exposure through medical school.

Furthermore, most of the respondents (45.9%, *n* = 259) who have had some exposure to CTS had no difference in opinion regarding the pursuit of a career in CTS after their experience, while others were motivated to further consider a career in CTS (37.9%, *n* = 214) or were deterred from CTS (13.8%, *n* = 78) after their exposure.

As students progressed through medical school, exposure to CTS appears to increase, with the lowest exposure in first year (18.5%, *n* = 62) and highest in the final year of medical school (44.5%, *n* = 69). In contrast, interest in pursuing CTS declined with progression through medical school, with 45.1% (*n* = 151) interested in first year compared to 16.8% (*n* = 26) of students by final year, potentially suggesting a negative correlation between CTS exposure and interest.

**Figure 1.**
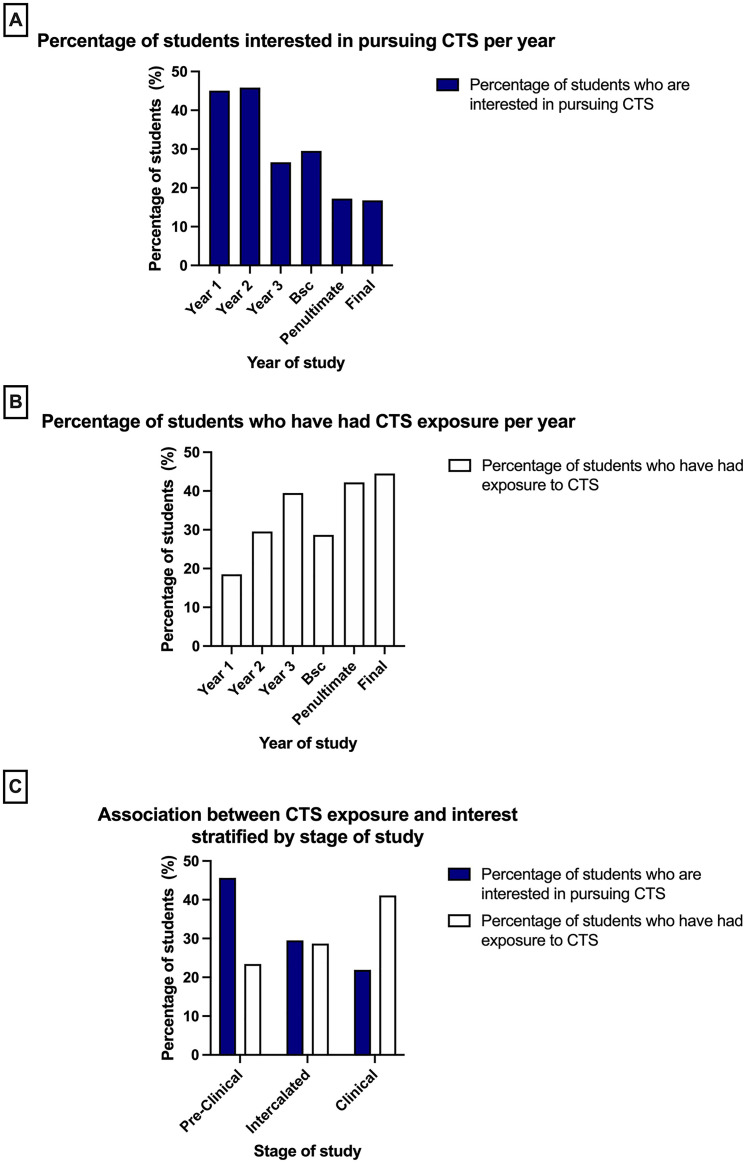
Students were asked whether they were interested in pursuing cardiothoracic surgery (CTS) and whether they had any exposure to CTS. **A-** Bar graph showing the percentage of medical students who are interested in pursuing CTS at every year of medical school. **B-** Bar graph showing the percentage of medical students who had exposure CTS at every year of medical school. **C-** Bar graph showing the level of interest and exposure students had at each stage in medical school (pre-clinical, intercalating and clinical).

Of the students who were interested in CTS, 40.5% (*n* = 213) had exposure to CTS, while 59.5% (*n* = 313) had no CTS exposure. Additionally, 14.6% (*n* = 77) of students interested in CTS were in their final or penultimate years, with the majority of those interested being pre-clinical students (Figure 1). Furthermore, of those students considering CTS as their top choice speciality, 57.5% had been exposed to CTS (*n* = 42). In those not interested in undertaking a career in CTS, 27.6% (*n* = 317) had previously considered CTS as a potential option.

### Student Perceptions of Potential Motivators and Barriers to CTS

**Figure 2.**
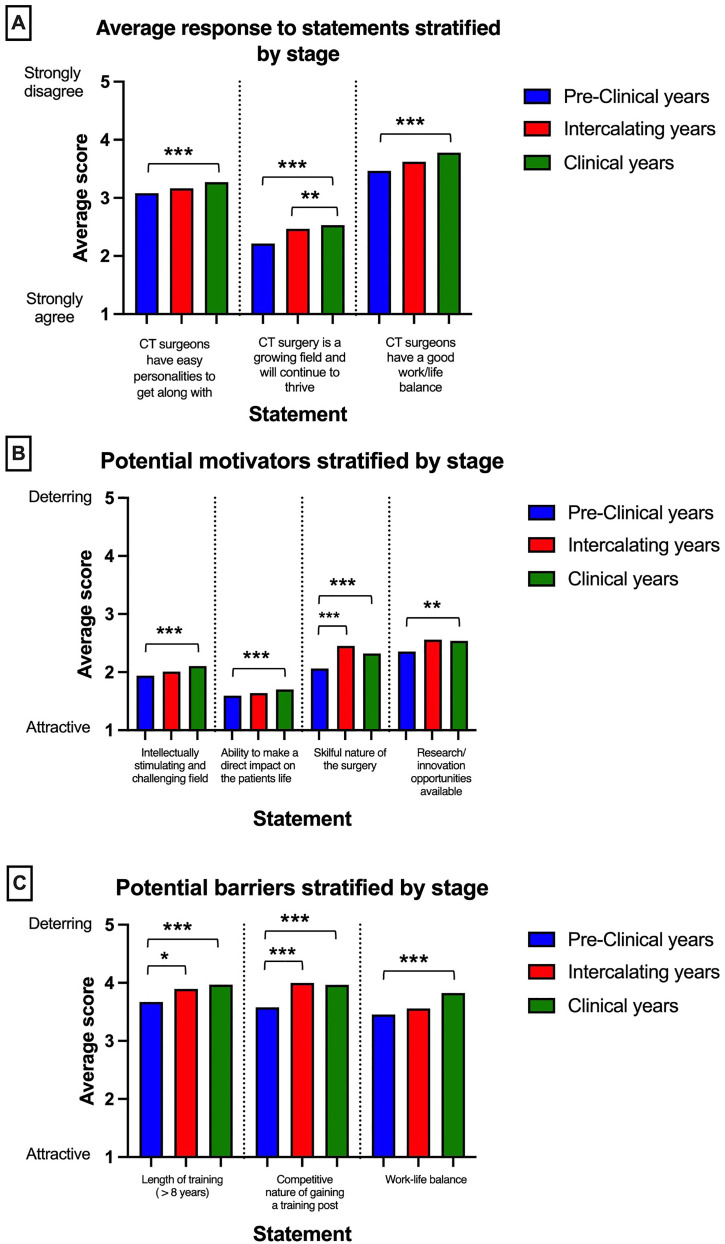
Students were asked to score statements from 1 to 5 regarding their views towards a career in CTS; results were stratified by the stage of their training (pre-clinical, intercalating and clinical). A Kruskal-Wallis test was conducted to investigate differences in CTS interest between the training stages, followed by a post-hoc Mann-Whitney test to see where any present significant difference lay. **A**- Bar graph showing mean scores from student ratings (1- strongly agree to 5- strongly disagree). **B**- Bar graph showing mean scores to statements that were found to be potential motivators (scores above 3 are considered deterring, while scores under 3 are considered attractive). **C**- Bar graph showing mean scores to statements that were found to be potential barriers. Asterisks denote significance: * p ≤ 0.05; p ≤ 0.01; p ≤ 0.001.

Survey respondents were asked to rate the following statements regarding a career in CTS on a 5-point Likert scale, with 1 representing strongly agree and 5 representing strongly disagree (Figure 2). The statements covered stereotypes of a career in CTS, their general perception of CTS as well as motivations and deterrents to choosing a career in CTS. Students were then asked to rate statements on a scale of attractiveness to the CTS pathway, with 1 being attractive and 5 being deterring. The responses were averaged and stratified based on year group as shown in Figure 2. Statements rated above 3 on average were considered as deterrents, while statements that scored less than 3 on average were considered as attractive factors for students (Figure 2). Responses varied significantly between students at different stages of medical school for all statements, with the significant differences primarily occurring between the clinical and pre-clinical years (Figure 2). Students in their pre-clinical years rated the below statements lower on average than other cohorts, indicating a more positive view of a career in CTS.

Overall, the most attractive factor to pursuing CTS was the ability to make a direct impact on patients’ lives (mean score 1.66) with 60.0% of students scoring it as 1 and 85.0% of respondents scoring it below 3, making it an attractive factor. The most deterring factor to students was the length of training with 63% of students rating it above 3 (mean score 3.85). Length of training was reported as the most deterring factor for pre-clinical students, whereas competitive nature of the getting into CTS was the most deterring factor for intercalating and clinical students.

### Gender, Interest and Exposure to CTS

A greater proportion of male respondents (38.0%, *n* = 235) showed interest in CTS than of females (27.6%, *n* = 289) or respondents of other genders (20%, *n* = 2). Further analysis was conducted into exposure of each gender to the speciality and the effect it had on the pursuit of CTS. We opted not to include other genders in this comparison as the number of respondents who identified as other gender were extremely small (*n* = 10).

**Figure 3.**
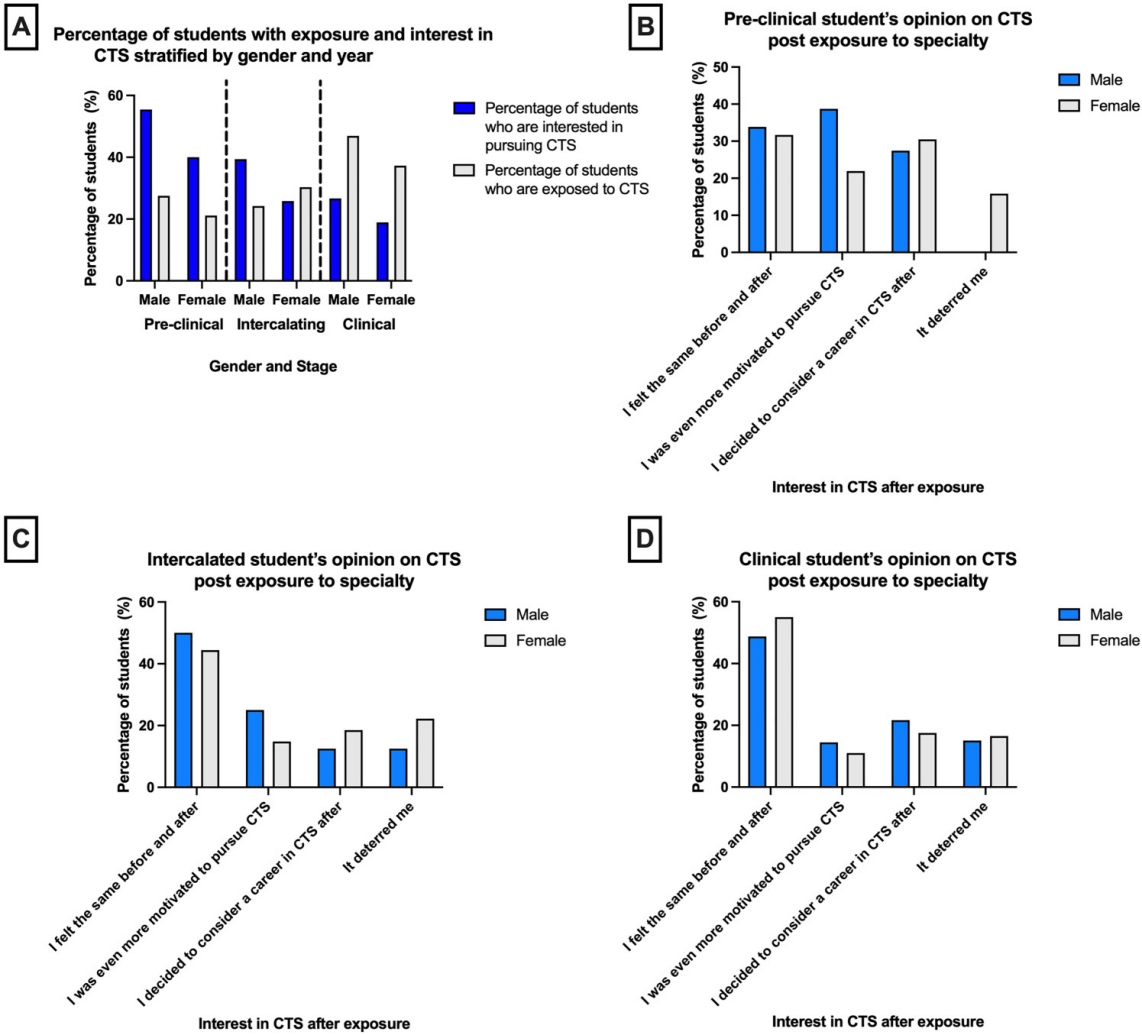
Medical students were asked whether they were interested in pursuing a career in CTS and whether they had exposure to it. **A-** Bar graph representing the percentage of students who had exposure to CTS (white) and the level of interest (blue) stratified by gender and stage at medical school. Students who did have exposure were asked how exposure influenced their decision with regards to seeking a career in CTS this was stratified by gender in all cases **B-** Bar graph showing the influence exposure had on pre-clinical students. **C-** Bar graph showing the influence exposure had on intercalating students. **D-** Bar graph showing the influence exposure had on clinical students.

We found that at pre-clinical and clinical stages, male students had greater exposure to CTS than their female counterparts (27.5% *vs.* 21.1% during pre-clinical stages and 47.1% *vs.* 37.1% during clinical stages, respectively). The percentage of student interest in CTS was also greater in men than women at every stage of medical school. This was despite the fact that exposure for female students was higher than that of male students during intercalation (Figure 3), although this might represent a small number of respondents.

When examining the effect of exposure on students’ opinions, a higher proportion of female students felt deterred from pursuing the speciality than their male counterparts at every educational stage.

### Gender and Perception of CTS

A difference between genders was found in the average responses to three statements in particular (Table 2). A larger proportion of female respondents found the length of training (65.4%, *p* = 0.0009) and competitive nature (67.7%, *p* < 0.0001) of gaining a CTS post to be a deterring factor more than their male counterparts (59.1% and 56.7% respectively). Furthermore, a larger percentage of males found the skilful nature of CTS surgery to be a motivating factor compared to female students, while both female and male students found the intellectual stimulations & challenges of the field to be similarly as motivating.

**Table 2.** A table outlining the proportions of scores towards perceived potential barriers and potential motivators for pursuing a career in CTS. Students were asked to score statements on a Likert scale from 1 to 5 regarding their views towards a career in CTS. These results were then stratified by gender (male and female). A score above 3 was considered a deterring factor, while a score below 3 was found to be a motivating factor.

**Panel A:** The percentage breakdown of scores to potential barrier statements stratified by gender. *^1^*P* = 0.0009, *^2^ *P* < 0.0001. **Panel B:** The percentage breakdown of scores to potential motivator statements stratified by gender. *^3^*P* = 0.0486, *^4^ *P* = 0.0005									
**A**: **Potential barriers**		Length of training (>8 years)*^1^		Competitive nature of gaining a training post*^2^			**Work-life balance**		
	Gender	Male	Female	Male	Female	Male		Female	
*Score*	***4 to 5*** *(deterrent)*	59.1%	65.4%	56.7%	67.7%	54.8%		57.2%	
	***3*** *(neutral)*	29.7%	27.8%	29.7%	24.7%	30.9%		28.5%	
	***1 to 2*** *(motivating)*	11.2%	6.8%	13.6%	7.6%	14.3%		14.3%	
**B: Potential motivators**		**Intellectually stimulating and challenging field**		Ability to make a direct impact on the patient's life *^3^		Skilful nature of the surgery*^4^		**Research/innovation opportunities available**	
	Gender	Male	Female	Male	Female	Male	female	Male	Female
*Score*	***4 to 5*** *(deterrent)*	11.5%	8.7%	7.8%	5.7%	15.7%	19.2%	16.3%	16.6%
	***3*** *(neutral)*	16.6%	18.7%	9.2%	8.1%	16.8%	20.1%	31.2%	34.1%
	***1 to 2*** *(motivating)*	71.9%	72.6%	83.0%	86.2%	67.5%	60.7%	52.5%	49.3%

## Discussion

### Interest and Exposure

As with any speciality, it is vital that a consistent stream of passionate and dedicated young doctors are recruited into the CTS career pathway to sustain and drive its evolution.^2^ Exploring the link between factors influencing student attitudes and their interest in CTS is important in order to adjust and improve current approaches.

With few CTS posts and a rigorous application process, gaining a CTS training position requires aspiring applicants to have strong portfolios which may include publications in peer-reviewed journals, high-ranking examination scores and demonstration of interest in CTS.^9^ To fulfil these requirements, early preparation during medical school is necessary. Understanding student perception of CTS and their attitudes towards different aspects of the career pathway is vital in order to stipulate recommendations to increase students’ access and interest in CTS early in medical school. Of those interested in pursuing CTS, only 6.3% were interested in thoracic surgery as a subspecialty. This study showed that up to 63% of students were deterred by the length of CTS training, currently 8 years. This presents the discussion of whether thoracic surgery should be considered as a separate surgical speciality, as seen in various countries, such as Brazil with total training length being as short as 5 years. More structured but shortened training periods may encourage students to pursue this speciality.^11,12^

A fundamental element of developing students’ interest in CTS is exposure to the speciality during medical school.^13,14^ This study found that with increased CTS exposure, interest in the specialty decreased. This was associated with a significant emphasis on deterring factors, particularly in medical students advancing from the pre-clinical to clinical stage. This is likely explained by two factors. Firstly, students who reported CTS exposure may have had negative clinical experiences or realised the challenges associated with the training and career pathway. Especially given the fact that CTS training is notorious for its physically and mentally demanding nature. Alternatively, the limited exposure to CTS in comparison with other specialities during clinical years of medical school may not provide enough opportunity for students to adequately consider the speciality as a future career. Importantly, only 40.5% of our participants had been exposed to CTS. This may be due to the greater focus during medical school to expose students to specialties that have more common presentations that a newly graduated doctor will have to manage independently. Yet certain specialities, such as general practice, play an important role in clinical education in UK medical schools, with the aim to encourage uptake in general practice.^15^ The increased focus and appealing presentation with potential benefits such as standard and flexible working hours, coupled with the financial benefits makes such careers more likely to attract increased attention and consideration from medical students. This increased focus on such specialties, however, reduces time spent within medical and surgical specialities such as CTS during clinical years of medical school.

Students with a positive experience of a speciality may be more likely to be interested and further commit to that speciality.^16^ It is therefore recommended to not only focus on increasing CTS exposure but importantly the quality of exposure in clinical practice to maintain interest.^16–18^ This may be difficult given the limited time shared between each speciality as students transition from pre-clinical to clinical years. However, exposure does not need to be clinical; one study exploring the effect of different types of CTS exposure on students’ interest found that non-clinical exposure was still associated with increased interest in pursuing CTS as a career choice.^19^ Other methods which have been shown to increase interest in CTS include simulation sessions with pre-clinical medical students and interactive workshops.^20,21^ However, one can argue that this does not take away from the deterring challenges of the speciality.

It is also important to consider the mindset and attitude of students at each stage of medical school. A study of medical student perceptions of a career in CTS in the US attributed the interest of younger medical students to the perceived openness in exploring career choices early on.^3^ This may also be the reason why pre-clinical students in our study were more likely to rate statements in the survey as more attractive or less deterrent. It is important to note that first year, pre-clinical students made up the largest proportion of respondents (20%), which may have resulted in a greater emphasis on positive attitudes within the results. It is therefore important that CTS engagement occurs at an early stage, during that period of openness, and is then reinforced with positive exposure to the specialty as the student progresses. In order to increase exposure of this speciality to students as early as possible we suggest medical schools host frequent speciality fairs within the academic year, during which students are exposed to all specialities available in medicine. This can include workshops and taster sessions to engage students, which have been shown to increase speciality interest.^4^ Furthermore, often in the first couple of years within the medical school curriculum the core concepts of human physiology are taught which encompasses teaching about the cardiovascular system. Medical school should aim to insert more workshops or tutorials on a topic within the speciality, also allowing for opportunities to discuss what a career within CTS will entail.

Moreover, we propose for there to be greater engagement between the national CTS society, medical schools and CTS medical school societies around the country by providing opportunities for students to get involved via shadowing experience, research placements etc

Although these points increase initial engagement, this does not detract from the challenges of the speciality such as length of training and work-life balance which students can gain a greater appreciation for during their clinical years, potentially contributing to the observed decline in interest in CTS as students progress through medical school.^22^ However, this reduction in interest may have been influenced by a large proportion of survey respondents being female (62.5%), who may have had further negative perceptions of these barriers due to the lack of representation support and work life balance. These factors have been shown to act as obstacles to women pursuing surgical careers in multiple cross-sectional studies.^23–25^ This warrants further analysis in our study.^26^

### Gender Differences in CTS

This study found that there was a difference between the proportion of female and male students interested in CTS (38% *vs.* 27.6% respectively). Female participants had a lower baseline interest in pursuing a CTS career which may indicate the need to increase female representation in the speciality.

Furthermore, this study found that a significantly larger proportion of female respondents found the length of training (*p* = 0.0009) and competitive nature (*p* < 0.0001) of gaining a CTS post to be a more deterring factor than their male counterparts. Additionally, the skilful nature of this speciality was found to be less of an attractive factor for a greater percentage of female respondents than their male counterparts (19.2% *vs.* 15.7% respectively), despite it being largely seen by both groups as a motivating factor. Notably, our results indicate that a greater proportion of female medical students were deterred from pursuing CTS at every stage of medical school than their male counterparts.

Reasons behind these different perceptions are varied and complex. Within the cohort of women interested in CTS, exposure appears to have deterred them from pursuing the specialty more so than their male counterparts. This could be related to more negative personal experiences of CTS or potentially the realisation of the challenges associated with the career. A major consideration when choosing a speciality is the work-life balance, which is shown to be a key differentiating factor between men and women's approach to speciality selection in various studies and commentaries by female surgeons.^27,28^ Additionally, some studies suggest that female medical students are discouraged to pursue the specialty due to the organisational culture and resulting impact on work-life balance, for example in relation to working hours.^29^ Hiryama *et al* added that the main barrier distinguishing female and male surgeons’ career progression is the culture supporting an inflexible training pathway, restricting permitted time out and subsequently leading to male domination in the surgical speciality.^30^ This is potentially exacerbated by the competitive nature of this speciality, where taking extended leaves due to family commitments^31^ may be feared by female medical students and trainees as a setback to their career, perhaps reducing overall interest in applying for CTS.^27,28,32^

In order to reduce barriers that prevent female applicants to the CTS pathway, change must be introduced at two levels: within medical schools and at an institutional level. At the former stage, mentorship has been widely found to be effective in increasing interest in surgical specialties.^33,34^ Optional mentorship schemes should therefore be made available to continue to engage and inspire those who are interested. This provides students with role models who can share experiences about the rewarding nature of their speciality alongside their methods of maintaining a work-life balance thus preventing the drop-in interest.

Implementing change at an institutional and organisational level is challenging but holds potential to have widespread positive impact on female perceptions and experience with CTS. More flexible training pathways, as seen in other specialties such as obstetrics and gynaecology, which also allow for less than full time training^35,36^ could help overcome this barrier. Moreover, there should be greater support, understanding and acceptance from senior colleagues for trainees who consider taking time out of training.

Although this study explores the potential deterrents and motivating factors for both genders, the nature of the questionnaire does not allow for full exploration of the origin of differences in perception between male and female medical students. Further research in this area allowing for free text-responses or focus groups is necessary in order to further examine this. This will enable us to further understand issues faced by prospective trainees, allowing for more specific recommendations to be implemented to encourage engagement in CTS.

## Limitations

There are several potential limitations that should be considered. Although we attempted to gauge students’ perceptions across all medical schools in the UK, self-selection bias may have occurred, despite efforts to reduce this by disseminating the questionnaire via social media, avoiding surgical/CTS specific groups. Due to the open and voluntary nature of the survey, being distributed via social media, calculating an accurate response rate was difficult. Additionally, the study's focus on CTS and its voluntary nature may have resulted in a greater number of students with an interest in CTS responding, resulting in response bias. This is a possible explanation for the greater proportion of students who stated they were considering a career in CTS in comparison to the proportion expected based on specialty application statistics.^37^ Use of the Likert-scale and lack of free text-questionnaires may have also exacerbated this bias. Obtaining a more widespread student view would reduce the response bias, requiring a larger sample size and a greater proportion of responses from each University cohort. Moreover, an aspect that was not evaluated in our study was the effect of financial renumeration on choosing CTS as a specialty, which has been shown to be a motivating factor and would be an interesting aspect to explore in future work.^38^

Although attempts were made to gain a representative sample by surveying medical schools across the UK, there was a large range in response numbers, possibly due to larger medical schools holding a greater proportion of responses. Additionally, in order to accurately assess the effect of exposure to CTS on students’ interest in the field, further questions must be asked about the exact nature of exposure, frequency and whether it was overall, a positive or negative experience, which our study did not assess.

Finally, our study looks at students’ exposure and perceptions of CTS at only one point in time; a longitudinal study would be a more accurate representation of changes in students’ beliefs over time, and would be useful in identifying the point at which differences in male and female perceptions begin to occur. Results from such a study could indicate measures/interventions required to improve medical students’ exposure to CTS during medical school.

## Conclusion

Many students displayed an interest in pursuing CTS. This study shows an interesting link between exposure and students’ interests in pursuing a career in CTS. Additionally, it provides a valuable insight for CTS trainers and educators into the challenges experienced by medical students and factors which are seen as motivators or barriers in pursuing CTS. We suggest that mentorship may be particularly important in closing the gap between male and female students regarding their interest in pursuing a CTS training post, as identified by our study.

Longitudinal studies encompassing focus groups will be required to fully understand students’ perceptions of this demanding speciality including the deterrents and attractive aspects of it. This would provide a more holistic view and allows for targeted recommendations for methods to improve exposure to CTS for medical students at the most appropriate time in their education.

## References

[1] Zakkar M Benedetto U Angelini GD , et al. Cardiothoracic surgery training in the United Kingdom. J Thorac Cardiovasc Surg. 2019;157(5):1948‐1955. doi:10.1016/j.jtcvs.2018.11.076. 30661815

[2] Westaby S Baig K De Silva R Unsworth-White J Pepper J . Recruitment to UK cardiothoracic surgery in the era of public outcome reporting. Eur J Cardio-thoracic Surg. 2015;47(4):679‐683. doi:10.1093/ejcts/ezu509. 25646396

[3] Specialty Advisory Committee; Society for Cardiothoracic Surgery in Great Britain and Ireland. UK Cardiothoracic Surgery Work Force Report 2019. Published 2019. Accessed February 20, 2020. https://scts.org/wp-content/uploads/2019/01/SCTS-workforce-report-2019.pdf

[4] Coyan GN Kilic A Gleason TG , et al. Medical student perceptions of a career in cardiothoracic surgery: results of an institutional survey. J Thorac Cardiovasc Surg. 2020;159(5):1906‐1912. doi:10.1016/j.jtcvs.2019.07.022. 31471086

[5] Bridgeman A Findlay R Devnani A , et al. Inspiring the next generation of cardiothoracic surgeons: an easily reproducible, sustainable event increases UK undergraduate interest in the specialty. Interact Cardiovasc Thorac Surg. 2016;22(1):106‐108. doi:10.1093/icvts/ivv280. 26467636

[6] Kesieme EB Abubakar U Olusoji O Inuwa IM Kefas J Anumenechi N . Factors affecting interest in cardiothoracic surgery among junior surgical residents in Nigeria. Cardiovasc J Afr. 2017;28(5):293‐297. doi:10.5830/CVJA-2017-004. 28252676 PMC5730681

[7] Bruce AN Battista A Plankey MW Johnson LB Blair Marshall M . Perceptions of gender-based discrimination during surgical training and practice. Med Educ Online. 2015;20(1):5-9. doi:10.3402/meo.v20.25923. PMC4317470 25652117

[8] Kristoffersson E Diderichsen S Verdonk P Lagro-Janssen T Hamberg K Andersson J . To select or be selected - gendered experiences in clinical training affect medical students’ specialty preferences. BMC Med Educ. 2018;18(268) 7-10. doi:10.1186/s12909-018-1361-5. PMC6245780 30453953

[9] Bowdish ME D’Agostino RS Thourani VH Schwann TA Krohn C Desai N , et al. STS adult cardiac surgery database: 2021 update on outcomes, quality, and research. Ann Thorac Surg. 2021;111(6):1770‐1780. 33794156 10.1016/j.athoracsur.2021.03.043

[10] [Internet]. Gmc-uk.org. 2021 [cited 10 November 2021]. https://www.gmc-uk.org/-/media/documents/gmc-somep-2020-referece-tables-about-medical-students_pdf-84718237.pdf

[11] Depypere LP Lerut AEMR . Thoracic surgical training in Europe: what has changed recently? Ann Transl Med. 2016;4(5):89. doi:10.21037/atm.2016.03.07. 27047948 PMC4791334

[12] Tedde M Petrere O Pinto Filho D Pereira S Monteiro R Sassaki A , et al. General thoracic surgery workforce: training, migration and practice profile in Brazil. Eur J Cardiothorac Surg. 2014;47(1):e19‐e24. 25391387 10.1093/ejcts/ezu411

[13] Marshall DC Salciccioli JD Walton SJ Pitkin J Shalhoub J Malietzis G . Medical student experience in surgery influences their career choices: a systematic review of the literature. J Surg Educ. 2015;72(3):438‐445. doi:10.1016/j.jsurg.2014.10.018. 25544332

[14] Al-Heeti KNM Nassar AK DeCorby K Winch J Reid S . The effect of general surgery clerkship rotation on the attitude of medical students towards general surgery as a future career. J Surg Educ. 2012;69(4):544‐549. doi:10.1016/j.jsurg.2012.04.005. 22677595

[15] Agha RA Papanikitas A Baum M Benjamin IS . The teaching of surgery in the undergraduate curriculum - reforms and results. Int J Surg. 2005;3(1):87‐92. doi:10.1016/j.ijsu.2005.03.017. 17462264

[16] Pianosi K Bethune C Hurley KF . Medical student career choice: a qualitative study of fourth-year medical students at Memorial University, Newfoundland. C Open. 2016;4(2):147‐152. doi:10.9778/cmajo.20150103. PMC4933642 27398357

[17] Vaporciyan AA Reed CE Erikson C , et al. Factors affecting interest in cardiothoracic surgery: Survey of North American general surgery residents. J Thorac Cardiovasc Surg. 2009;137(5):1054‐1062. doi:10.1016/j.jtcvs.2009.03.044. 19379966

[18] Kilcoyne MF Do-Nguyen CC Han JJ , et al. Clinical exposure to cardiothoracic surgery for medical students and general surgery residents. J Surg Educ. 2020;77(6):1646‐1653. doi:10.1016/j.jsurg.2020.05.017. 32522562

[19] Gasparini M Jayakumar S Ayton S Nardini MN Dunning JD . Medical student exposure to cardiothoracic surgery in the United Kingdom. Interact Cardiovasc Thorac Surg. 2019;29(2):173‐178. doi:10.1093/icvts/ivz038. 30879049

[20] George J Combellack T Lopez-Marco A , et al. Winning hearts and minds: inspiring medical students into cardiothoracic surgery through highly interactive workshops. J Surg Educ. 2017;74(2):372‐376. doi:10.1016/j.jsurg.2016.10.002. 27789191

[21] Lou X Enter D Sheen L , et al. Sustained supervised practice on a coronary anastomosis simulator increases medical student interest in surgery, unsupervised practice does not. Ann Thorac Surg. 2013;95(6):2057‐2063. doi:10.1016/j.athoracsur.2013.02.045. 23706428

[22] Preece R Ben-David E Rasul S Yatham S . Are we losing future talent? A national survey of UK medical student interest and perceptions of cardiothoracic surgery. Interact Cardiovasc Thorac Surg. 2018;27(4):525‐529. doi:10.1093/icvts/ivy119. 29672686

[23] Brundage S Lucci A Miller C Azizzadeh A Spain D Kozar R . Potential targets to encourage a surgical career. J Am Coll Surg. 2005;200(6):946‐953. 15922210 10.1016/j.jamcollsurg.2005.02.033

[24] Bellini M Graham Y Hayes C Zakeri R Parks R Papalois V . A woman’s place is in theatre: women’s perceptions and experiences of working in surgery from the Association of Surgeons of Great Britain and Ireland women in surgery working group. BMJ Open. 2019;9(1):e024349. 10.1136/bmjopen-2018-024349 PMC6326292 30617103

[25] Brown C Harries R Abdelrahman T Thomas C Pollitt M Lewis W . Surgical gender gap: a curriculum concordance and career vector perspective. Postgrad Med J. 2018;94(1115):483‐488. 30355590 10.1136/postgradmedj-2017-135437

[26] Weissman C Zisk-Rony RY Schroeder JE , et al. Medical specialty considerations by medical students early in their clinical experience. Isr J Health Policy Res. 2012;1(1):13. doi:10.1186/2045-4015-1-13. 22913658 PMC3424828

[27] Antonoff MB Brown LM . Work-life balance: the female cardiothoracic surgeon’s perspective. J Thorac Cardiovasc Surg. 2015;150(6):1416‐1421. doi:10.1016/j.jtcvs.2015.09.057. 26478231

[28] Abbasi K . Understanding career barriers for women in surgery. J R Soc Med. 2018;111(9):307. doi:10.1177/0141076818800119. 30226098 PMC6146343

[29] Backhus LM Fann BE Hui DS Cooke DT Berfield KS Moffatt-Bruce SD . Culture of safety and gender inclusion in cardiothoracic surgery. Ann Thorac Surg. 2018;106(4):951‐958. doi:10.1016/j.athoracsur.2018.07.011. 30120943

[30] Hirayama M Fernando S . Organisational barriers to and facilitators for female surgeons’ career progression: a systematic review. J R Soc Med. 2018;111(9):324‐334. doi:10.1177/0141076818790661. 30175935 PMC6146338

[31] Liang R Dornan T Nestel D . Why do women leave surgical training? A qualitative and feminist study. Lancet. 2019;393(10171):541‐549. doi:10.1016/S0140-6736(18)32612-6. 30739689

[32] Marks IH Diaz A Keem M , et al. Barriers to women entering surgical careers: a global study into medical student perceptions. World J Surg. 2020;44:37‐44. doi:10.1007/s00268-019-05199-1. 31616970

[33] Drolet BC Sangisetty S Mulvaney PM Ryder BA Cioffi WG . A mentorship-based preclinical elective increases exposure, confidence, and interest in surgery. Am J Surg. 2014;207(2):179‐186. doi:10.1016/j.amjsurg.2013.07.031. 24269035

[34] O’Connor MI . Medical school experiences shape women students’ interest in orthopaedic surgery. Clin Orthop Relat Res. 2016;474(9):1967‐1972. doi:10.1007/s11999-016-4830-3. 27084717 PMC4965370

[35] Henry A Clements S Kingston A Abbott J . In search of work/life balance: trainee perspectives on part-time obstetrics and gynaecology specialist training. BMC Res Notes. 2012;5(19):4. doi:10.1186/1756-0500-5-19. PMC3276423 22233566

[36] Health Education England. Appendix B: Approaches to Flexible Working – Evidence from the individual specialties: Obstetrics & Gynaecology and Paediatrics. Published 2016. Accessed February 20, 2021. https://www.hee.nhs.uk/sites/default/files/documents/Appendix B - Medical Royal Colleges’ Feedback on Flexibility_0.pdf

[37] Specialty Training – Health Education England. 2020 Competition Ratios. Published 2020. Accessed January 30, 2021. https://specialtytraining.hee.nhs.uk/Portals/1/2020 Competition Ratios.pdf

[38] Győrffy Z Birkás E Sándor I . Career motivation and burnout among medical students in Hungary - could altruism be a protection factor? BMC Med Educ. 2016;16(1):4. 10.1186/s12909-016-0690-5 PMC4950634 27430960

